# Appropriate DevR (DosR)-Mediated Signaling Determines Transcriptional Response, Hypoxic Viability and Virulence of *Mycobacterium tuberculosis*

**DOI:** 10.1371/journal.pone.0035847

**Published:** 2012-04-26

**Authors:** Shyamasree De Majumdar, Atul Vashist, Sakshi Dhingra, Rajesh Gupta, Alka Singh, Vijay K. Challu, V. D. Ramanathan, Prahlad Kumar, Jaya Sivaswami Tyagi

**Affiliations:** 1 Department of Biotechnology, All India Institute of Medical Sciences, New Delhi, India; 2 National Tuberculosis Institute, Bangalore, India; 3 Department of Pathology, Tuberculosis Research Centre, Chennai, India; Johns Hopkins University School of Medicine, United States of America

## Abstract

**Background:**

The DevR(DosR) regulon is implicated in hypoxic adaptation and virulence of *Mycobacterium tuberculosis*. The present study was designed to decipher the impact of perturbation in DevR-mediated signaling on these properties.

**Methodology/Principal Findings:**

*M. tb* complemented (Comp) strains expressing different levels of DevR were constructed in Mut1* background (expressing DevR N-terminal domain in fusion with AphI (DevR_N_-Kan) and in Mut2Δ*devR* background (deletion mutant). They were compared for their hypoxia adaptation and virulence properties. Diverse phenotypes were noted; basal level expression (∼5.3±2.3 µM) when induced to levels equivalent to WT levels (∼25.8±9.3 µM) was associated with robust DevR regulon induction and hypoxic adaptation (Comp 9* and 10*), whereas low-level expression (detectable at transcript level) as in Comp 11* and Comp15 was associated with an adaptation defect. Intermediate-level expression (∼3.3±1.2 µM) partially restored hypoxic adaptation functions in Comp2, but not in Comp1* bacteria that co-expressed DevR_N_-Kan. Comp* strains in Mut1* background also exhibited diverse virulence phenotypes; high/very low-level DevR expression was associated with virulence whereas intermediate-level expression was associated with low virulence. Transcription profiling and gene expression analysis revealed up-regulation of the phosphate starvation response (PSR) in Mut1* and Comp11* bacteria, but not in WT/Mut2Δ*devR*/other Comp strains, indicating a plasticity in expression pathways that is determined by the magnitude of signaling perturbation through DevR_N_-Kan.

**Conclusions/Significance:**

A minimum DevR concentration of ∼3.3±1.2 µM (as in Comp2 bacteria) is required to support HspX expression in the standing culture hypoxia model. The relative intracellular concentrations of DevR and DevR_N_-Kan appear to be critical for determining dormancy regulon induction, hypoxic adaptation and virulence. Dysregulated DevR_N_-Kan-mediated signaling selectively triggers the PSR in bacteria expressing no/very low level of DevR. Our findings illustrate the important role of appropriate two-component- mediated signaling in pathogen physiology and the resilience of bacteria when such signaling is perturbed.

## Introduction

*Mycobacterium tuberculosis* (*M. tb*) remains a major human pathogen causing widespread disease and mortality. It is estimated that an infected individual has a 5–10% risk of developing TB over his/her lifetime [1], although this scenario shows regional variation that is influenced to a significant extent by HIV infection [2]. The synergistic effect of the two diseases and outbreaks of drug-resistant TB (MDR-TB, XDR-TB) are significant hindrances in the treatment of this killer disease. Another major challenge in controlling tuberculosis is the lack of suitable anti-tubercular therapy against non-replicating persistent bacteria [3]. Consequently, there has been considerable effort in understanding mycobacterial persistence and dormancy in the recent years, in order to devise strategies targeted towards their control and elimination [4], [5].

The interaction of *M. tb* with the host is dynamic and complex during the multiple phases of its intracellular adaptation and involves the participation of numerous regulatory networks [6]. Two-component systems play a central role in bacterial adaptation by regulating a spectrum of physiological processes ranging from nutrient uptake to virulence. DevR-DevS (also called as DosR-DosS) is the best characterized two-component system of *M. tb*. DevR is implicated in the adaptation of *M. tb* to potential host-derived signals such as hypoxia, nitric oxide, carbon monoxide or ascorbic acid [7]–[10] and also in *M. tb* virulence [11]–[15]. DevR is positively auto regulated under activating conditions [16] and it induces the expression of ∼47 genes that collectively constitute the DevR regulon [7], [17].

DevR is a typical response regulator belonging to the NarL subfamily and it contains a N-terminal phosphorylation domain and a C-terminal DNA binding domain [18]. The requirement of phosphorylation to induce DevR regulon gene expression is well established [16], [19], [20]. Numerous studies have demonstrated the important role of DevR during the physiological adaptation of *M. tb* to hypoxia [7], [15], [21]–[25]. We recently showed that co-expression of DevR and DevR_N_-Aph fusion protein (DevR_N_-Kan) in *M. tb* Comp1* bacteria led to defective hypoxic adaptation and attenuation of virulence [15]. DevR_N_-Kan protein functions as an inhibitor of DevR signalling and we suggested that the *level* of activated DevR may be a critical determinant of DevR function. In the present study, we tested this hypothesis by analyzing *M. tb* complemented (Comp) strains engineered to express DevR at either high, intermediate or low levels through the use of promoters of different strengths. Comp* strains that expressed DevR at high levels (∼33±6 µM) were restored in both DevR regulon induction and hypoxia survival functions. However, Comp* strains that expressed DevR at low levels failed in both responses. In Comp* strains with intermediate DevR levels such as Comp1*, DevR_N_-Kan competed effectively for the activating phosphosignal resulting in a partial defect in hypoxic adaptation. Intriguingly, strains expressing either a high or a low level of DevR/no DevR, were virulent in the guinea pig model of infection while intermediate-level expression was associated with attenuation. The possible underlying mechanism and scope for plasticity in DevR-mediated phosphosignalling are discussed.

## Results

### Inhibitory action of DevR_N_-Kan is modulated by the level of DevR under hypoxia

**Figure 1:**
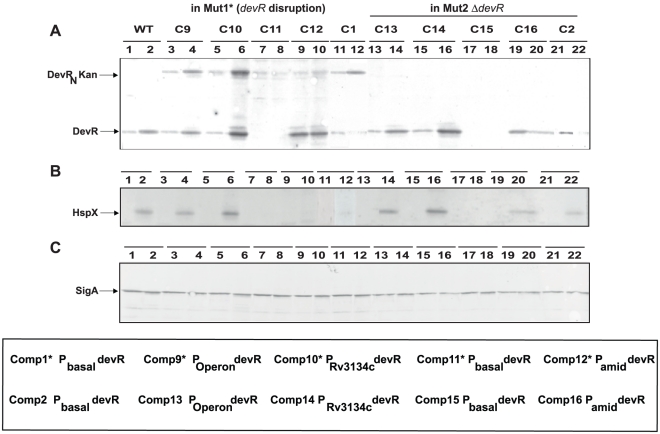
Immunoblot analysis. Lysates of *M. tb* strains were electrophoresed and subjected to SDS-PAGE and immunoblot analysis using polyclonal antibodies against (**A**) DevR, (**B**) HspX and (**C**) SigA. In all panels: lanes 1–2, WT; lanes 3–4, Comp9*; lanes 5–6, Comp10*; lanes 7–8, Comp11*; lanes 9–10, Comp12*; lanes 11–12, Comp1*; lanes 13–14, Comp13; lanes 15–16, Comp14; lanes 17–18, Comp15; lanes19–20, Comp16 and lanes 21–22, Comp2. Lanes 1, 3, 5, 7, 9, 11, 13, 15, 17, 19 and 21 represent aerobic cultures; lanes 2, 4, 6, 8, 10, 12, 14, 16, 18, 20 and 22 represent 5 days standing hypoxic cultures. Representative blots from 2 to 4 independent experiments are shown.

Various *M. tb* Comp strains that were constructed as described (Tables 1 and 2) were monitored for HspX expression as a measure of DevR regulon induction in response to hypoxia. As expected, DevR and HspX protein expression was induced (∼4-fold and ∼7-fold, respectively) in hypoxia-adapted WT cultures (Figure 1, lanes 1–2). Among Comp* strains (in Mut1* background that co-express DevR and DevR_N_-Kan proteins), the induction response is comparable to that of WT where DevR is expressed from the inducible promoter (Figure 1, lanes 3–4 and 5–6, in Comp9* and Comp10*, respectively), whereas only a modest induction of HspX was observable in Comp1* which expresses DevR from a low copy number plasmid under its basal promoter (Figure 1, lanes 11–12). However, HspX expression was below the limit of detection by western blotting in Comp11* and Comp15 (Figure 1, lanes 7–8 and 17–18) but detectable by RNA measurements (see below). The low-level expression of DevR in Comp11* and Comp15 strains is attributed to DevR being expressed from a single chromosomally integrated copy of the gene under its basal promoter.

**Table 1:** Plasmids used in this study.

Plasmids	Description	Reference
pFPV27	*E. coli-Mycobacterium* shuttle plasmid with promoterless gfp, km^r^	[52]
pDSDevR	*devR* coding sequence and 327 bp upstream region cloned in pFPV27 (low copy number plasmid), hyg^r^	[12]
pOperon	*Rv3134c-devRS* operon promoter (−608 to +998) cloned in pFPV27, hyg^r^	[16]
p3134c-1	*Rv3134c* promoter (−608 to +90) cloned in pFPV27, hyg^r^	[16]
pJFR19	3-kb amidase promoter cloned in mycobacterial integrative vector pMV306H, hyg^r^	[53]
pMG85	*devR* coding region cloned downstream of amidase promoter in integrative vector pJFR19, hyg^r^	[54]
pSM P_Operon_ devR	*Rv3134c-devRS* operon promoter (−608 to +998) replaces amidase promoter in pMG85, hyg^r^	This study
pSM P*_Rv3134c_* devR	*Rv3134c* promoter (−608 to +90) replaces amidase promoter in pMG85, hyg^r^	This study
pSM P*_devR_* devR	*devR* promoter (−327 to −1) replaces amidase promoter in pMG85, hyg^r^	This study
pKKNKan	pET-28a- based plasmid for overexpression of DevR_N_-Kan fusion protein, Kan^r^	[15]
pAVDevR	pET-28a- based plasmid for overexpression of intact DevR protein, Kan^r^	[15]

**Table 2:** *Mycobacterium tuberculosis* strains used in this study.

*M. tb* strain	Description	Expression		Reference
		DevR	DevR_N_-Kan	
WT	H37Rv	+	−	[12]
Mut1* (*devR* disruption)	*devR* disruption mutant expressing Dev_N_-Kan	−	+	[12]
Mut2 Δ*devR*	*devR* deletion mutant	−	−	[11]
Comp1* P _basal_ *devR*	Mut1* complemented with plasmid pDSDevR	+	+	[12]
Comp9* P_Operon_ *devR*	Mut1* complemented with integrated pSM P_Operon_ devR	+	+	This study
Comp10* P_Rv3134c_ *devR*	Mut1* complemented with integrated pSM P*_Rv3134c_* devR	+	+	This study
Comp11* P _basal_ *devR*	Mut1* complemented with integrated pSM P*_devR_* devR	+	+	This study
Comp12* P_amid_ *devR*	Mut1*complemented with integrated pMG85	+	+	This study
Comp2 P _basal_ *devR*	Mut2 Δ*devR* complemented with pDSDevR	+	−	[15]
Comp13 P_Operon_ *devR*	Mut2 Δ*devR* complemented with integrated pSM P_Operon_ devR	+	−	This study
Comp14 P_Rv3134c_ *devR*	Mut2 Δ*devR* complemented with integrated pSM P*_Rv3134c_* devR	+	−	This study
Comp15 P _basal_ *devR*	Mut2 Δ*devR* complemented with integrated pSM P*_devR_* devR	+	−	This study
Comp16 P_amid_ *devR*	Mut2 Δ*devR* complemented with integrated pMG85	+	−	This study

The precise role of promoter strength in DevR regulon gene expression was assessed in Comp strains generated in Mut2Δ*devR* background (without interference from DevR_N_-Kan). The HspX induction response in these Comp strains was similar to those in Mut1* background *only* when DevR was expressed from the inducible promoter (Figure 1, lanes 14 and 16). Notably, inducible HspX expression was partially restored in Comp2 and Comp16 strains (Figure 1, lanes 22 and 20), but not in Comp1* and Comp12* bacteria (equivalent strains in Mut1* background). Consistent with our previous observations [15], DevR was not upregulated in Comp1* and Comp2 strains under hypoxia; rather its level declined over time (Figure 1 lanes 11–12, 21–22). However, truncated DevR_N_-Kan was induced over the 5 day period of hypoxic incubation in Comp1* bacteria (Figure 1, lanes 11–12). These results suggest that expression level of DevR is dependent on the strength of the promoter which in turn modulates the inhibitory action of DevR_N_-Kan.

**Figure 2:**
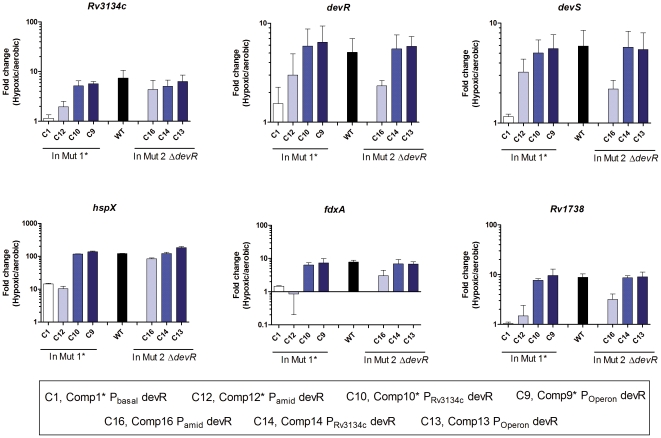
Expression analysis of selected DevR regulon transcripts. Various *M. tb* Comp strains were grown to an A_595_ of 0.2–0.3 under aerobic conditions and kept standing for 5 days (hypoxic cultures). Gene expression was assessed in Comp strains generated in Mut1* and Mut2Δ*devR* backgrounds by qRT Reverse transcriptase-PCR analysis. Fold change in target gene expression in hypoxic vs. aerobic cultures was calculated from normalized transcript levels with respect to 16S rRNA. Mean fold change ± SD determined from three independent cultures is shown.

At the transcriptional level also, as expected, regulon genes transcription was induced in WT cultures under hypoxia (Figure 2). Among the Comp* strains that co-express DevR_N_-Kan and DevR, induction of the regulon was restored to nearly WT levels only in Comp9* and Comp10* strains which support inducible DevR expression (Figure 2). A modest induction of select regulon transcripts was noted in Comp11* bacteria expressing DevR from the basal promoter (Figure S1), whereas an intermediate level of induction was observed in Comp1* and Comp12* bacteria that express DevR from the basal/constitutive amidase promoter (Figure 2). For example, *hspX* RNA expression was powerfully induced by ∼120-fold in hypoxic cultures of WT, Comp9* and Comp10* bacteria vs. a relatively modest induction of 10- to 14-fold in Comp1* and Comp12* and <2-fold induction in Comp11* bacteria.

Regulon gene expression in Comp strains expressing only intact DevR (no DevR_N_-Kan) in Mut2Δ*devR* background was assessed simultaneously to decipher the role of promoter strength. The induction response was equivalent in Comp strains constructed in both Mut1* and Mut2Δ*devR* backgrounds *only* when DevR is expressed from the inducible or basal promoter (Figure 2). In contrast, Comp16 strain (expressing DevR from the amidase promoter) exhibited a fairly robust induction response for *hspX*, *fdxA* and *Rv1738* (84-, 3- and 3- fold respectively) compared to markedly lower induction response (10-, 1-, 1.5- fold respectively) in Comp12* bacteria (Mut1* background). DevR_N_-Kan mediated inhibition was also evident in Comp11* vs. Comp15 bacteria (Figure S1). These findings collectively indicate that both the extent of hypoxic gene induction and the degree of inhibition by DevR_N_-Kan are modulated by the level of DevR.

### Quantification of the intracellular concentration of DevR

**Figure 3:**
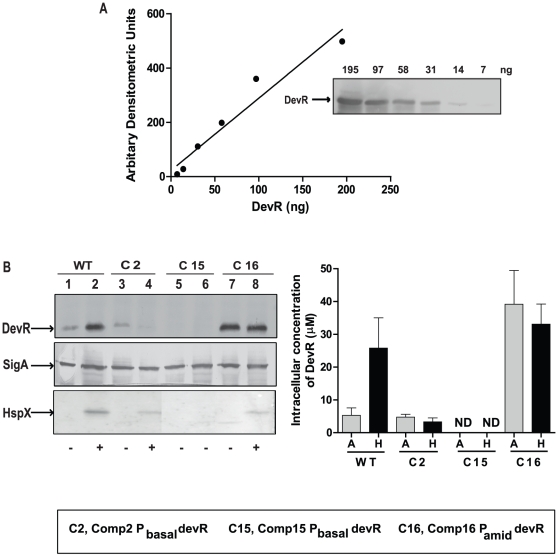
Estimation of intracellular concentration of DevR. (**A**) A standard curve for DevR protein was generated using purified DevR protein and quantitative Western blotting. (**B**) *M. tb* lysates (15 µg protein) were electrophoresed and subjected to immunoblot analysis in parallel with purified DevR protein using polyclonal antibodies to DevR, SigA (not shown) or HspX (lower panel). ‘−’ and ‘+’ indicate absence and presence of HspX expression. In all panels, lanes 1, 3, 5 and 7 represent aerobic cultures and lanes 2, 4, 6 and 8 represent hypoxic cultures. The immunostained blots were scanned and analyzed with software Quantity One. The data are the averages of two independent determinations. ND, not detected.

The intracellular concentration of DevR was estimated in select *M. tb* strains by quantitative immunoblotting as described [26] to determine the critical concentration required for the regulon induction response. Purified His_6_-DevR protein was used as a standard in the Western blot assays (Figure 3A). The intracellular concentration of DevR was estimated as ∼5.3±2.3 µM under aerobic (basal) conditions and it rose ∼5-fold to ∼25.8±9 µM under hypoxia in WT cultures (Figure 3B). In contrast, the concentration of DevR was lower (∼3.3 µM) in Comp2 and not detectable in Comp15 bacteria. The *hspX* promoter is the earliest promoter to be induced under hypoxia in a DevR-dependent manner and it is induced even prior to the operon promoter [17] and therefore its expression was monitored in select Comp strains to determine the minimum DevR concentration that is sufficient to support its induction. From quantitative immunoblot analysis, it is estimated that a minimum DevR concentration of ∼3.3±1.2 µM (as in Comp2 bacteria) is required to support HspX expression in the standing culture hypoxia model. Importantly, HspX induction occurs on activation of *existing* DevR molecules (∼3.3 µM concentration in Comp2) and not in aerobic WT bacteria which actually express DevR at slightly higher concentrations (∼5.3 µM). This clearly establishes that phosphorylation is essential for generating ‘active’ DevR protein. This study also demonstrates that an increase in *devR* transcripts due to autoregulation is associated with ∼5-fold increase in intracellular DevR protein concentration under hypoxia.

**Figure 4:**
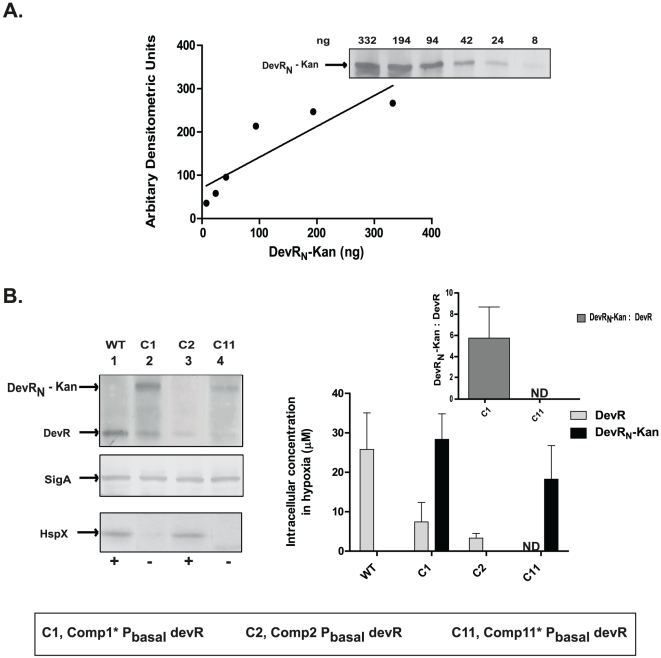
Estimation of intracellular DevR_N_-Kan and DevR concentration in *M. tb* under hypoxia. (**A**) A standard curve was generated for DevR_N_-Kan protein as for DevR protein. (**B**) *M.tb* lysates prepared from hypoxic cultures (15 µg) were electrophoresed and analyzed by immunoblotting as described in the legend to Figure 3. ‘−’ and ‘+’ indicate absence and presence of HspX expression. Inset, Ratio of normalized DevR_N_-Kan and DevR proteins in various Comp strains. The values shown are the averages of two independent determinations.

Because the inhibitory action of DevR_N_-Kan appears to be modulated by the intracellular concentration of DevR protein, the intracellular concentration of DevR_N_-Kan in select *M. tb* Comp* strains was also estimated (Figure 4A, B). A DevR_N_-Kan∶DevR ratio of ≥5.8 as in Comp1* bacteria interfered with robust HspX induction in this strain whereas a lower concentration of DevR in Comp2 bacteria (3.3±1.2 uM) supported HspX induction in the absence of DevR_N_-Kan inhibitor (Figure 4B). These findings suggest that both DevR_N_-Kan∶DevR ratio as well as DevR concentration play an important role in regulating DevR regulon gene expression in *M. tb* strains that express this inhibitor.

### DevR expression level is critical for hypoxic viability of *M. tb*

**Figure 5:**
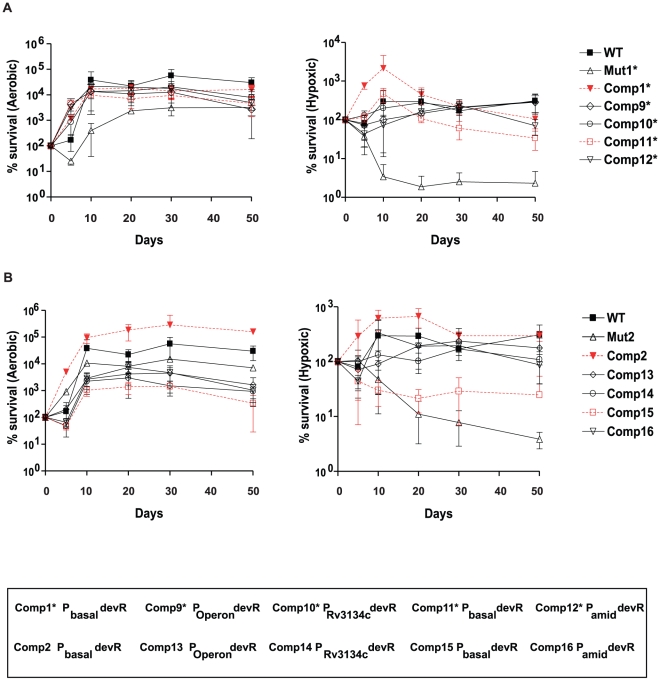
Survival of H37Rv (WT), mutant (Mut1* and Mut2Δ*devR*) and various complemented *M. tb* strains. The survival of various *M. tb* strains was assessed under aerobic and hypoxic conditions over a period of 50 days. The mean CFU ± SD determined from three independent cultures is shown as % survival with respect to CFU on day zero.

The various *M. tb* strains were analyzed for their comparative survival fitness properties under hypoxia (Figure 5). WT bacteria sustained well and the initial load remained viable on day 50. However, Mut1* and Mut2Δ*devR* strains were defective in hypoxic adaptation; their viability decreased from day 5 onwards (the earliest time point when bacteria were sampled) and only ∼2% of Mut1* and ∼3% of Mut2Δ*devR* bacteria were viable on day 50 (Figure 5). Those Comp strains that express a high level of DevR (from the inducible/constitutive promoter) adapted well under hypoxia; on day 50 their viability ranged between 132 to 80% relative to initial CFU observed on day 0. However, under hypoxic conditions, Comp1* and Comp2 grew more rapidly than WT bacteria but *only* during the first 10 days and thereafter the viability decreased and on day 50, ∼5% of Comp1* and ∼48% of Comp2 bacteria were viable relative to maximum CFU observed on day 10. Interestingly, Comp11* and Comp15 bacteria, that express a very low level of DevR (undetectable by western blotting), exhibited a less severe hypoxic survival defect as compared to the parental Mut strains; ∼33% and ∼24% viability of Comp11* and Comp15 bacteria in comparison to only 3% and 5% viability, respectively of Mut1* and Mut2Δ*devR* bacteria on day 50 with respect to the initial bacterial load. These observations, on one hand provide functional evidence for DevR expression in these Comp strains and on the other hand, confirm the inhibitory activity of DevR_N_-Kan. All the strains showed a similar survival profile over a time period of 50 days under aerobic conditions. These findings establish that not only DevR is essential but its level is critical for the hypoxic adaptation and survival of *M. tuberculosis*.

### The level of DevR expression modulates virulence of *M. tb* in guinea pig

**Figure 6:**
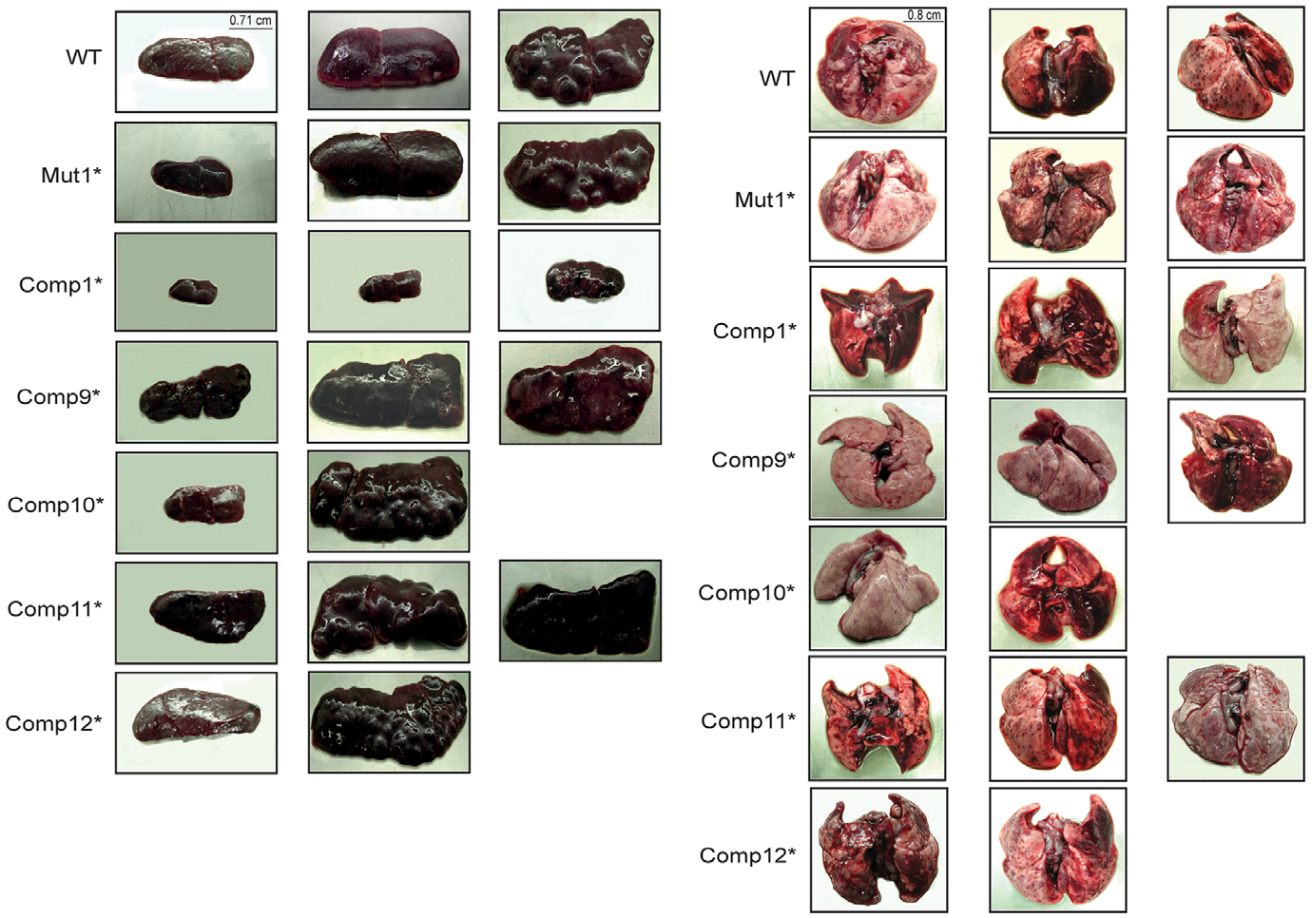
Pictorial representation of spleen and lungs. The figure depicts representative photographs of lungs and spleen of animals infected with different *M. tb* strains euthanized at 6, 10 and 13 weeks post-infection.

**Figure 7:**
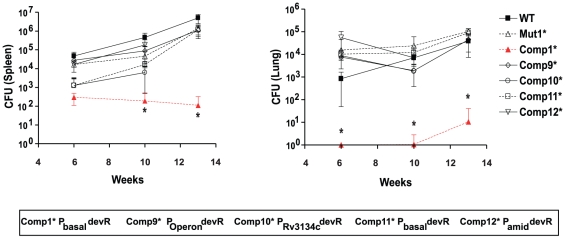
Bacterial load in spleens and lungs of *M. tb*-infected guinea pigs. CFU are expressed as Mean ± SD. [*, (P<0.05) in comparison to WT]. CFU of lung is that of the right lower lobe.

The virulence properties of various passaged *M. tb* strains including H37Rv (WT), *devR* mutant (Mut1*) and various DevR_N_-Kan inhibitor-expressing Comp* strains were compared in the guinea pig model of virulence as described [15]. In this subcutaneous infection model, bacterial implantation in the lung and other organs by dissemination requires time. At 6 weeks, the earliest time point studied, nearly similar numbers of lesions were visually scored for the WT, Mut1* and Comp9* -Comp11* strains. The visual score of the Comp12* group was higher whereas fewer lesions were scored in the Comp1* group (P<0.05 in comparison to WT, Table S1). Splenic enlargement was noted in all infected animals with the exception of Comp1*-infected guinea pigs (Table S1, Figure 6). At 13 weeks the weight of the animals infected with the Comp1* strain was comparatively higher (statistically significant, P<0.05) than that of the animals infected with WT or Mut1* or other Comp* strains (Figure S2). The bacterial loads in lung and spleen were also lower in the Comp1* group (P<0.05 in comparison to WT, Figure 7).

For the evaluation of disease progression, later time points in infection were assessed. An almost identical trend of visual scores, though of a higher magnitude, correlating with disease progression, was observed at 10 weeks and 13 weeks post-infection for all the groups except Comp1* (P<0.05). It is also noteworthy that the visible tubercles in lungs of Mut1*- and Comp11*-infected animals were comparable or even slightly more in comparison to WT- infected animals. The spleens and lungs were significantly inflamed in animals infected with all *M. tb* strains with the exception of Comp1*. Bacterial multiplication continued between 6 and 13 weeks post-infection in both spleen and lung organs (Figure 7). Interestingly, the Comp1* strain was significantly attenuated at all the time points. Based on the comparative assessment of various Comp* strains in the guinea pig model, it is evident that the level of DevR expression profoundly influences the virulence properties of these strains. Thus, while a high level of DevR expression (as in Comp9*, Comp10*, Comp12*) is associated with virulence, a moderate level of DevR expression (Comp1*) leads to a low virulence phenotype and a very low level of DevR expression (as in Comp11*) restores the virulence phenotype. The difference in the infecting dose as the reason for the low virulence phenotype of Comp1* is ruled out as an equal number of viable bacteria were injected. The growth defect of Comp1* in guinea pig is likely due to the decreased infectivity of Comp1* and its gradual clearing during the course of infection as described earlier [15].

### Mut1* bacteria exhibit a constitutive phosphate starvation response

The virulent phenotype of the Mut1* strain in the guinea pig model suggests that either DevR is not essential for *M. tb* virulence or that virulence may be mediated through an alternative pathway in Mut1* bacteria. With a view to obtain insights into the possible molecular mechanisms underlying the virulence of Mut1* strain, genome-wide transcriptome analysis of Mut1*, Comp1* and WT strains was performed.

**Figure 8:**
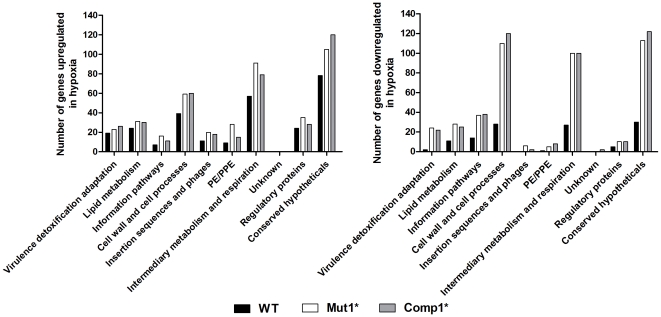
Genes significantly upregulated or downregulated during hypoxia in WT, Mut1* and Comp1* strains. Fold change 5 day/0 day (Hypoxia/Aerobic) was calculated. Significant differential regulation: ≥1.5-fold induction, ≤0.66-fold repression (P≤0.05).

Hypoxia led to an induction of 270, 297 and 389 genes in the WT, Mut1* and Comp1* strains, respectively. Forty genes of the DevR/DosR regulon were amongst the genes that were induced in the WT strain. Of the remaining genes induced under hypoxia in the WT strain, 66 genes belonged to the Enduring Hypoxic Response (EHR) category as described [13]. Thirty nine genes of the EHR category were up-regulated in all three strains indicating their induction in a DevR independent manner as reported [13]. A total of 435 and 449 genes were downregulated in Mut1* and Comp1* as compared to 118 genes in WT bacteria under hypoxia (Figure 8). The *nuoABEFIJKLMN* genes that encode subunits of NADH dehydrogenase, the ubiquinol–cytochrome C complex (*qcrABC*), and ATP synthase (*atpABDEFGH*) that are involved in normal aerobic growth were either repressed or not induced under hypoxia in all the 3 strains. Their repression likely reflects reduced aerobic respiration during bacteriostasis under hypoxic conditions. Genes belonging to the intermediary metabolism and respiration category were downregulated to a greater extent in Mut1* and Comp1* bacteria under hypoxia as compared to the WT bacteria (100 and 100 genes vs. 27 genes, respectively). In Mut1* and Comp1* bacteria more genes for amino acid biosynthesis (12 and 7 genes vs. 7 in WT), nucleotide metabolism (5 and 1 genes vs. 1 in WT), lipid biosynthesis (19 and 16 genes, respectively vs. 11 genes in WT), were repressed. Likewise, in the Mut1* and Comp1* bacteria, more genes (110 and 117 genes, respectively vs. 28 genes in WT) belonging to the cell wall and cell processes category (including cellular transport genes) were downregulated. Twenty three of the ribosomal protein coding genes in Mut1* were repressed compared to 9 and 16 genes, respectively, in the WT and Comp1* strains. Overall, the repression of genes belonging to intermediary metabolism, lipid metabolism, information pathways, cell wall and cell processes functional categories in the Mut1* and Comp1* bacteria may be the reason for their defective hypoxic adaptability. An overall comparison of our data to the previously published microarray-generated gene lists shows partial overlaps (Table S2). However, the differences in the number of genes up/downregulated in various publications are possibly due to the different models used for developing hypoxia.

**Figure 9:**
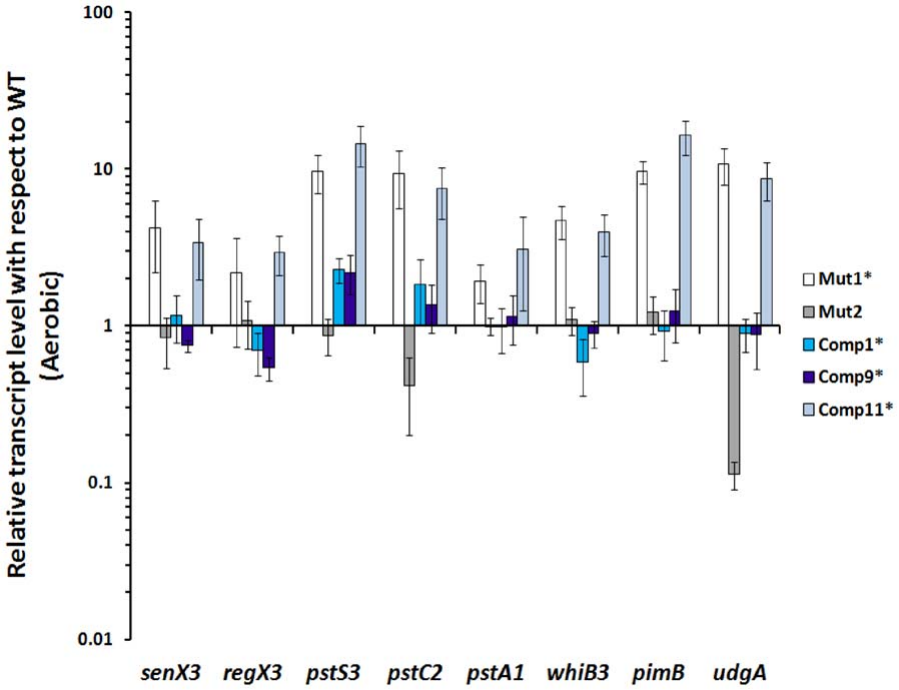
Differentially expressed genes in Mut1* bacteria. Relative aerobic transcript levels (Mean ± SD) of PSR genes in Mut1* and Comp1* strains with respect to that in WT bacteria were calculated from C_t_ values determined by RT reverse transcriptase-PCR analysis. Data from three independent cultures is shown.

Intriguingly, several genes were selectively upregulated under both aerobic and hypoxic conditions in Mut1* bacilli in comparison to WT and Comp1* strains. These included those genes encoding the two-component system *senX3*-*regX3*, phosphate transport operon *pstS3*-*pstC2*- *pstA1*, *whiB3*, *pimB*, *udgA*, PPE 25, PE18, PPE51 etc., Table 3, Figure S3). The differential expression of these genes in Mut1* bacilli was confirmed by qRT reverse transcriptase-PCR analysis (Figure 9). Interestingly, all these genes were also reported to be upregulated in response to phosphate starvation in *M. tb* and to constitute the phosphate starvation response [27]. Our results suggested a possible role for DevR_N_-Kan inhibitor in eliciting the phosphate starvation response (PSR) in Mut1* bacteria. Therefore, additional *M. tb* strains, namely, Mut2Δ*devR*, Comp9* and Comp11* were analysed to obtain further insight. Interestingly, the PSR genes were found to be up-regulated in Comp11* bacteria (Figure 9), which like Mut1* bacteria express a high level of DevR_N_-Kan protein. The pivotal role of DevR_N_-Kan in eliciting a PSR is supported by the absence of this response in Mut2Δ*devR* bacteria which does not express DevR_N_-Kan protein or in Comp9* bacteria or Comp1* bacteria where DevR_N_-Kan-mediated phosphosignaling appears to be neutralized by co-expression of DevR. We conclude that the PSR genes were up-regulated in strains that express a high level of DevR_N_-Kan protein and a very low level of DevR (Comp11*) or no DevR at all (Mut1*). Note that upregulation of PSR genes occurs in spite of growth in phosphate-sufficient medium. The combined results of the guinea pig model and expression analysis implicate the PSR genes in an alternative pathway for virulence in Mut1* and Comp11* strains. Further studies are required to elucidate the precise determinants governing plasticity of the PSR response in the presence of DevR_N_-Kan inhibitor.

**Table 3:** Genes upregulated in Mut1* under aerobic and hypoxic conditions.

Rv #	Gene Name	Mut1* Aer	Comp1* Aer	Mut1* Hyp	Comp1* Hyp
*Rv0322*	*udgA*	6.76	1.03	1.89	0.66
*Rv0490*	*senX3*	3.21	1.01	1.68	1.09
*Rv0491*	*regX3*	3.31	1.19	2.47	1.58
*Rv0557*	*pimB*	4.75	0.83	2.80	0.78
*Rv0784*	*Rv0784*	31.09	1.38	5.92	0.75
*Rv0785*	*Rv0785*	3.98	1.11	3.45	0.80
*Rv0928*	*pstS3*	7.13	0.96	3.13	0.68
*Rv0929*	*pstC2*	7.18	1.36	3.69	1.03
*Rv0930*	*pstA1*	4.64	1.14	2.69	1.01
*Rv1419*	*Rv1419*	1.85	0.88	1.91	1.36
*Rv1787*	*PPE25*	8.46	0.80	4.51	1.05
*Rv1788*	*PE18*	7.84	0.96	3.74	0.67
*Rv3027c*	*Rv3027c*	3.17	1.01	2.33	1.30
*Rv3046c*	*Rv3046c*	2.26	1.21	1.65	1.34
*Rv3416*	*whiB3*	7.14	0.64	3.52	1.15

Geometric mean of normalized signal intensities in Mut1* and Comp1* bacteria expressed in aerobic and hypoxic cultures with respect to that in aerobic and hypoxic WT cultures, respectively. Genes upregulated in Mut1* are listed (Geometric mean of normalized signal intensity ≥1.8). ‘Aer’ and ‘Hyp’ refer to aerobic (0 day) and hypoxic (5 day) conditions.

## Discussion

In the present study, ten complemented *M. tb* strains that varied in DevR expression level were constructed in two *devR* mutant backgrounds and they were assessed for their hypoxic induction response, hypoxic survival and virulence properties. The DevR_N_-Kan inhibitor was observed to modulate the hypoxic response in a DevR concentration-dependent manner. For the first time, the intracellular level of DevR protein in WT bacteria was determined and estimated to be ∼5 µM (average ∼3×10^3^ molecules/cell) under basal (aerobic) conditions. Its expression was induced ∼5-fold under hypoxia to attain an intracellular concentration of ∼26 µM (average ∼2×10^4^ molecules/cell). The increase in intracellular protein concentration of DevR is consistent with the previously reported autoregulation of the *Rv3134c-devRS* operon at the transcriptional level [16]. The elevated concentration of DevR under hypoxia is comparable with that of CtrA transcriptional regulator in *Caulobacter crescentus* which ranges between 10–30 µM [28] and that of PhoP response regulator in *Salmonella enterica* which increases from a basal concentration of ∼2.7 µM to ∼11 µM under conditions of Mg^2+^ limitation [29].We recently established the presence of at least 47 sites at 19 chromosomal locations that bind to DevR to induce hypoxic expression of DevR/DosR regulon genes [17]. The accumulation of DevR under hypoxia observed in the present study is consistent with an increased requirement of this regulator for interaction at these binding sites. The combined results of expression analysis and viability measurements of various *M. tb* strains indicate that the intracellular concentration of DevR is crucial for bacterial adaptaion under hypoxia. Strains expressing DevR at equivalent levels as the WT strain supported both a robust induction response and viability under prolonged hypoxia. However, strains expressing a very low level of DevR were defective in both functions. These findings underscore the importance of DevR-mediated adaptive responses and are consistent with previous reports that DevR is essential for bacterial survival under hypoxia [15], [21], [30]. Our study also establishes that DevR concentration is not limiting in WT bacteria to induce the DevR regulon under aerobic conditions. Rather, the observed HspX induction in Comp2 bacteria implies that a lower intracellular DevR concentration (∼3.3 µM) can trigger induction under hypoxia and establishes the essentiality of phosphorylation for generating ‘active’ DevR protein.

Quantitative western blotting analysis also indicated that a DevR_N_-Kan/DevR ratio of ≥∼5.75 leads to an abrogation of regulon expression (as in Comp1* and Comp11*). A possible explanation is that DevR_N_-Kan may compete with DevR for the activating phosphosignal as occurs *in vitro* [15]. The structure adopted by DevR_N_-Kan may support its phosphorylation in aerobic cultures unlike DevR whose closed structure was postulated to undergo helical rearrangements upon phosphorylation in order to generate the active DNA binding species [31]. Moreover, phosphorylation of isolated N-terminal domains of response regulators DrrB, DrrD and MtrA (of OmpR/PhoB family) is enhanced compared to that of the corresponding full-length proteins [32]. Thus the basis of the expression defect in Comp1* bacteria is likely to be DevR_N_-Kan-mediated disregulation of phosphosignaling. The analysis of complemented strains in a complete *devR* deletion background (Mut2Δ*devR*) lends credence to this hypothesis. For instance, the partial restoration of the induction response and viability under hypoxia was noted in Comp2, but not in Comp1* bacteria. Likewise, a comparison of the strains expressing DevR from a constitutive promoter (Comp12* and Comp16 bacteria) revealed that Comp12 (in Mut1* background) but not Comp16 bacteria (in Mut2Δ*devR* background) was defective in the hypoxia induction response. A similar observation has been made in *B. subtilis* where the N-terminal domain of Spo0A response regulator acts as a competitor of Spo0A function [33].

Some noteworthy phenotypes were observed in the guinea pig virulence model in the present study; intermediate-level expression of DevR in Comp1* bacteria was associated with low virulence whereas very low-level expression as in Comp11 *or high- level expression in Comp9*, Comp10*, Comp12* bacteria were associated with bacterial virulence. Thus the expression level of DevR appears to be critical for determining virulence and is supported by published reports which suggest that *M. tb* strains that express DevR at different levels do exhibit varying degrees of virulence. Clinical isolates belonging to the sub lineages of the W/Beijing family were found to constitutively overexpress many DevR regulon genes suggesting a role for elevated expression on intracellular adaptation [34]. The significantly increased virulence of Comp12* strain compared to WT bacteria at 6 weeks post-infection may be attributed to the constitutive expression of DevR in this strain and associated pre-adaptation of Comp12* bacteria within the host. Although several studies have highlighted the role of DevR in bacterial adaptation and virulence [7], [11]–[15], [35]–[40], it is intriguing that either expression of DevR_N_-Kan alone (as in Mut1*) or its co-expression with low level of DevR (as in Comp11* bacteria) was associated with the virulence phenotype. These results highlight the plasticity of signaling in *M. tb* and underscore the importance of appropriate DevR-mediated signaling in determining virulence outcomes of *M. tb*.

An interesting finding of transcriptome analysis was the upregulation of *regX3-senX3* two-component system and PSR genes in Mut1* bacteria. Notably, these genes were also upregulated in Comp11* bacteria but not in Mut2Δ*devR* strain. For the reason that it lacks the DNA binding domain, we do not believe that DevR_N_-Kan can ‘cross-activate’ a different TCS regulon in the conventional way. However, the DevR_N_-Kan module appears to be involved in the ‘activation’ of *regX3-senX3* in some other way. Since (a) DevR_N_-Kan is efficiently phosphorylated in vitro and competes with DevR for the phosphosignal [15] and (b) DevR_N_-Kan is well expressed within bacteria, a possible mechanism is that phosphorylation of DevR_N_-Kan through low molecular weight phosphor donors such as acetyl phosphate or by non-cognate sensor kinases may deplete intracellular phosphate stores resulting in phosphate starvation conditions. Further investigation is required to understand the role of DevR_N_-Kan in generating phosphate starved conditions. Phosphate limitation is the signal for activation of SenX3-RegX3 in *M. smegmatis* [41]. Therefore it is possible that DevR_N_-Kan functions as a phosphate sink and leads to ‘phosphate starvation’ conditions to activate SenX3-RegX3 in *M. tb* as well. Taking together these findings, the mechanism of SenX3-RegX3 regulon induction in *only* some strains may relate to the relative concentration of DevR_N_-Kan and DevR proteins. Mut1* expresses only DevR_N_-Kan protein while Comp11* has a high ratio of DevR_N_-Kan/DevR. Since the PSR genes are induced only in these two strains, it is likely that an ‘adequate’ ratio is relevant. On the other hand, other Comp strains (Comp9*, 10*) express DevR at higher levels Therefore the DevR_N_-Kan/DevR ratios are lower and an adequate concentration of DevR would support DevR regulon induction in these strains and preclude DevR_N_-Kan function as a phosphate sink.

The phosphate starvation response was recently characterized in *M. tb* and includes *senX3*-*regX3*, phosphate transport operon *pstS3-C2-A1*, *whiB3*, *ppk1* genes etc. [27]. The upregulation of the *pstS3-pstC2-pstA1* operon and not of the 2 other putative Pst operons (*pknD-pstS2* and *pstB-pstS1-pstC1-pstA2*) in the present study is suggestive of ‘phosphate limiting’ conditions within Mut1* and Comp11* bacteria. This is consistent with the occurrence of phosphate limiting conditions and the requirement for *pstS3-pstC2-pstA1* genes during *M. tb* growth in vivo [42]. RegX3 has been previously reported to regulate *ppk1* expression, which is known to regulate (p)ppGpp synthesis through *mprA*-*sigE*-*relA* pathway [43]. Upregulation of the *ppk1*, *sigE* and *relA* genes but not of *mprA* in Mut1* bacilli suggest that this may lead to induction of the stringent response in this strain through a MprA-independent pathway. Several genes belonging to the PE and PPE family such as those encoding PPE 25, PE 18 and PPE 51 were also upregulated in Mut1* and Comp11* bacteria. Members of this family have been shown to have antigenic potential [44], [45]. Therefore, the upregulation of PE/PPE genes may play a role in the enhanced intracellular survival of these strains. *In vivo* and *ex-vivo* studies of *senX3* and *regX3* mutant strains has highlighted the role of RegX3 in survival and persistence of *M. tb* and have suggested that *M. tb* is exposed to a P_i_-limited environment in mammalian lungs and that SenX3-RegX3 may play an important role in *M. tb* virulence [11], [27], [46]. The observed upregulation of this two-component system in Mut1* and Comp11* bacteria may facilitate their preferential multiplication in guinea pig lungs compared to the WT strain. WhiB3, whose transcripts were also up-regulated, is a transcription factor that is essential for survival and persistence of *M. tb* [47], [48]. Therefore, WhiB3 may also confer a survival advantage to Mut1* and Comp11* bacilli.

In summary, the upregulation of the PSR genes may help Mut1* and Comp11* bacteria to adapt better to stress within the host and thereby contribute to their virulence in guinea pigs. While the *level* of DevR appears to be critical for hypoxic survival, regulon induction and virulence, our findings also point towards the crucial role of *appropriate* DevR signaling in determining the outcome of the bacterial adaptation response. This study reveals hitherto to unknown plasticity in DevR-mediated phosphosignalling. It has implications in understanding the consequences of perturbing sigalling pathways in *M. tb* and may provide novel strategies for intervention of *Mycobacterium tuberculosis* adaptation in vivo.

## Materials and Methods

### Ethics statement

Due approval was accorded by the Institutional Animal Ethics Committee, NTI, Bangalore prior to the animal experiments.

### Construction of plasmids

The plasmids and strains used in this study are described in Tables 1 and 2, respectively. DNA fragments carrying different *M. tb* promoters were excised out of plasmids pOperon, p3134c-1 and pDSdevR using appropriate restriction enzymes. The Operon promoter and the *Rv3134c* promoter were then cloned in the integrative plasmid vector pMG85 by replacing the amidase promoter to generate pSM P_operon_ devR and pSM P*_Rv3134c_* devR. The *devR* coding sequences along with its own upstream promoter were cloned into the pJFR19 integrative vector by replacement of the amidase promoter to generate pSM P*_devR_* devR (Table 1). All the plasmids were verified by DNA sequencing.

### Construction of *M. tb* strains

The constructed plasmids (Table 1) were electroporated into *M. tb* Mut1* and Mut2Δ*devR* strains to generate various complemented strains (Comp series) that are described in Table 2. The identity of the strains was confirmed by PCR analysis. ‘*’ is added to all strains that express DevR_N_-Kan protein (Mut1*, Comp1* etc.).

### Preparation of passaged bacilli

All experiments were performed with guinea pig-passaged *M. tb* WT, Mut1* and Comp* strains unless mentioned otherwise. For passaging, frozen stocks of laboratory cultured bacilli (at −80°C) were thawed, resuspended in PBS and ∼5×10^6^ CFU were injected subcutaneously into guinea pigs. Passaged bacteria were recovered from guinea pigs at 6 weeks post infection by plating spleen homogenates on Middlebrook (MB) 7H11 agar with OADC. Bacterial scrapings were cultured once in MB 7H9 medium containing Albumin Dextrose Complex (ADC) and stored at −80°C for further use. The passaged strains were verified by PCR analysis.

### Assessment of viability of *M. tb* strains *in vitro*

Frozen passaged *M. tb* stocks were sub cultured twice or thrice in Dubos medium containing ADC and 0.1% Tween 80 to logarithmic phase (A_595_ ∼0.4). The cultures were diluted to A_595_ of 0.005 and 10 ml aliquots were dispensed in 50 ml tubes and grown either with shaking at 220 rpm (aerobic setup) or kept standing in 15 ml tubes (hypoxic setup). Cultures were sampled once only from separate tubes dedicated for each time point of the hypoxia set up. Bacterial CFU at defined time points was estimated by plating serial dilutions in duplicate on MB 7H11 agar containing ADC and incubating the plates at 37°C for 6 weeks.

### Quantitation of bacterial gene expression

Various *M. tb* strains were cultured as described above with shaking at 220 rpm from an initial A_595_ of 0/025(100 ml culture in a 500 ml flask). At A_595_ ∼0.2–0.3, a 50 ml aliquot was centrifuged immediately (aerobic culture) and the remaining culture aliquot was kept standing (10 ml in 50 ml tubes) at 37°C (hypoxic culture). RNA was isolated using TRI reagent (Molecular Research Center, USA) and purified using RNeasy mini column (Qiagen, Germany). Reverse transcription was performed with random hexamer primers and cDNA High capacity Reverse Transcriptase kit (ABI, USA). *M. tb* mRNAs were quantitated by real-time PCR using gene-specific primers (Supplementary Table S3) in a MyIQ Single colour detection thermal cycler (Biorad Inc.).

### Expression analysis by immunoblotting

*M. tb* strains were grown to logarithmic phase as described above, diluted to A_595_ of 0.025 and grown with shaking to an A_595_ of 0.3. A culture aliquot was immediately harvested by centrifugation (aerobic culture). For hypoxic cultures, 10 ml aliquots of aerobic cultures were dispensed into 50 ml screw-capped tubes and kept standing for 5 days. Lysates were prepared as described [49] from two to four independent cultures at each condition and 10 or 15 µg protein was analyzed by 12.5% SDS-PAGE and immunoblotting using rabbit polyclonal antisera as described [25]. Anti-SigA antibody was a generous gift from Dr. T.S. Balganesh (AstraZeneca, Bangalore).

For measurement of intracellular concentrations of DevR and DevR_N_-Kan proteins, a standard curve for each of the purified proteins was prepared (based on triplicate measurements) using an appropriate range (198 ng to 7 ng for DevR and 332 ng to 8 ng for DevR_N_-Kan) as described [26]. *M. tb* lysates were analysed at least thrice in parallel with the purified proteins. After immunostaining, the blots were scanned and then intensities of the stained DevR and DevR_N_-Kan bands were quantitated (Quantity One software, Biorad, USA). The signal intensities derived from SigA were used to normalize the signal intensities from DevR and DevR_N_-Kan in each lysate. Calculations of intracellular concentrations were based on various parameters, namely, efficiency of *M. tb* lysis (assumed to be ∼85% based on laboratory experience), reported total average mass of protein/cell (150 fg), a cellular volume of 0.94 fl [50] and a molecular mass of 23.2 kDa and 45 kDa for DevR and DevR_N_-Kan, respectively.

### Guinea pig virulence assay

Guinea pigs (4–8 animals per group) were infected by subcutaneous route with passaged *M. tb* strains in phosphate buffered saline (approx 5×10^6^ viable organisms per animal) for 6, 10 and 13 weeks. The virulence assay was performed as described [12]. In this model, bacteria spread to the lungs and spleen from the site of injection (thigh). Briefly, at the time of sacrifice, internal organs were examined for visually scorable lesions in spleen, liver, lung, inoculation site and its draining lymph nodes as described [51]. Lungs and spleens were transferred to selective Kirchner's liquid medium for CFU determination [12]. The spleens and right lower lobes of lungs were individually homogenized in dedicated homogenizers and serial dilutions were plated in duplicate on MB 7H11 agar containing OADC and also on LJ slopes. The colonies were counted after 6 weeks of incubation at 37°C and the identity of the recovered bacilli was confirmed by PCR analysis. The statistical significance of the differences between the various strains for different parameters was determined using the Mann-Whitney test.

### Transcriptome comparison

RNA was extracted from 3–5 replicate cultures of each *M. tb* strain grown as described above (in the section ‘Mut1* bacteria exhibit a constitutive phosphate starvation response’). Total RNA of 1 µg was polyadenylated and cDNA was synthesized from the polyadenylated RNA samples. Cy3/Cy5 labelled cRNA was synthesized and amplified using Agilent Low RNA Input Amplification Kit PLUS, Two-Color. Genomic DNA of *M. tb* H37Rv was labeled using Agilent Genomic DNA labeling kit PLUS. The labeled RNA samples were hybridized against the labeled genomic DNA on Agilent custom made 8×15 K *M. tb* array with 60 mer probes using the Agilent platform (Genotypic Technology, Bangalore, India).

### Microarray data extraction and analysis

The slides were scanned using the Agilent Genepix scanner and data was extracted using the Agilent Feature Extraction Software. Single color analysis was performed by splitting the Two Color hybridized samples into single channel. Quantile normalization was done using GeneSpring GX software using the recommended Per Chip and Per Gene normalization. Normalized signal intensity of samples was compared with that of the control *i.e* Cy3/Cy5 labeled H37Rv genomic DNA for each gene among 3–5 biological replicates. Genes were considered to be upregulated for values ≥1.5 and down regulated for values ≤0.66) under aerobic (0 day) or hypoxic (5 day) conditions (P-value≤0.05). Raw and fully annotated microarray data has been deposited with NCBI and the GEO Accession No. GSE30264 (www.ncbi.nlm.nih.gov/geo/query/acc.cgi?acc=GSE30264)

## Supporting Information

Figure S1 Induction of select DevR regulon genes in Comp11* and Comp15. Fold change in *hspX* and *Rv1738* expression in hypoxic vs. aerobic cultures in Comp11* and Comp15 strains was calculated from normalized transcript levels with respect to 16S rRNA. Mean fold change ± SD determined from three independent cultures is shown.
(TIF)

Figure S2 **Comparison of gross body weights of** ***M. tb*** **infected guinea pigs.** Gross body weights expressed as (Mean ± SD). [*, statistically significant (P<0.05) in comparison to WT].
(EPS)

Figure S3 **Differential gene expression in** ***M. tb*** **strains.** Heat map depicting the fold change in differential gene expression (log base 2) in *M. tb* strains (2–5 biological replicates).
(TIF)

Table S1 **Virulence of passaged** ***M. tuberculosis*** **strains.**
(DOCX)

Table S2 **Comparison of our results with previously published microarray analysis. (A)** ***M. tb*** **WT (H37Rv), (B)** ***M. tb dosR*** **(** ***devR*** **) mutant.**
(DOC)

Table S3 **Primers used for Real Time Reverse transcriptase-PCR analysis.**
(DOCX)
