# Supplementary figures and images for: Appropriate DevR (DosR)-Mediated Signaling Determines Transcriptional Response, Hypoxic Viability and Virulence of Mycobacterium tuberculosis
Source: PLoS One. 2012 Apr 26;7(4):e35847. doi: 10.1371/journal.pone.0035847 (PMC3338549; doi:10.1371/journal.pone.0035847)

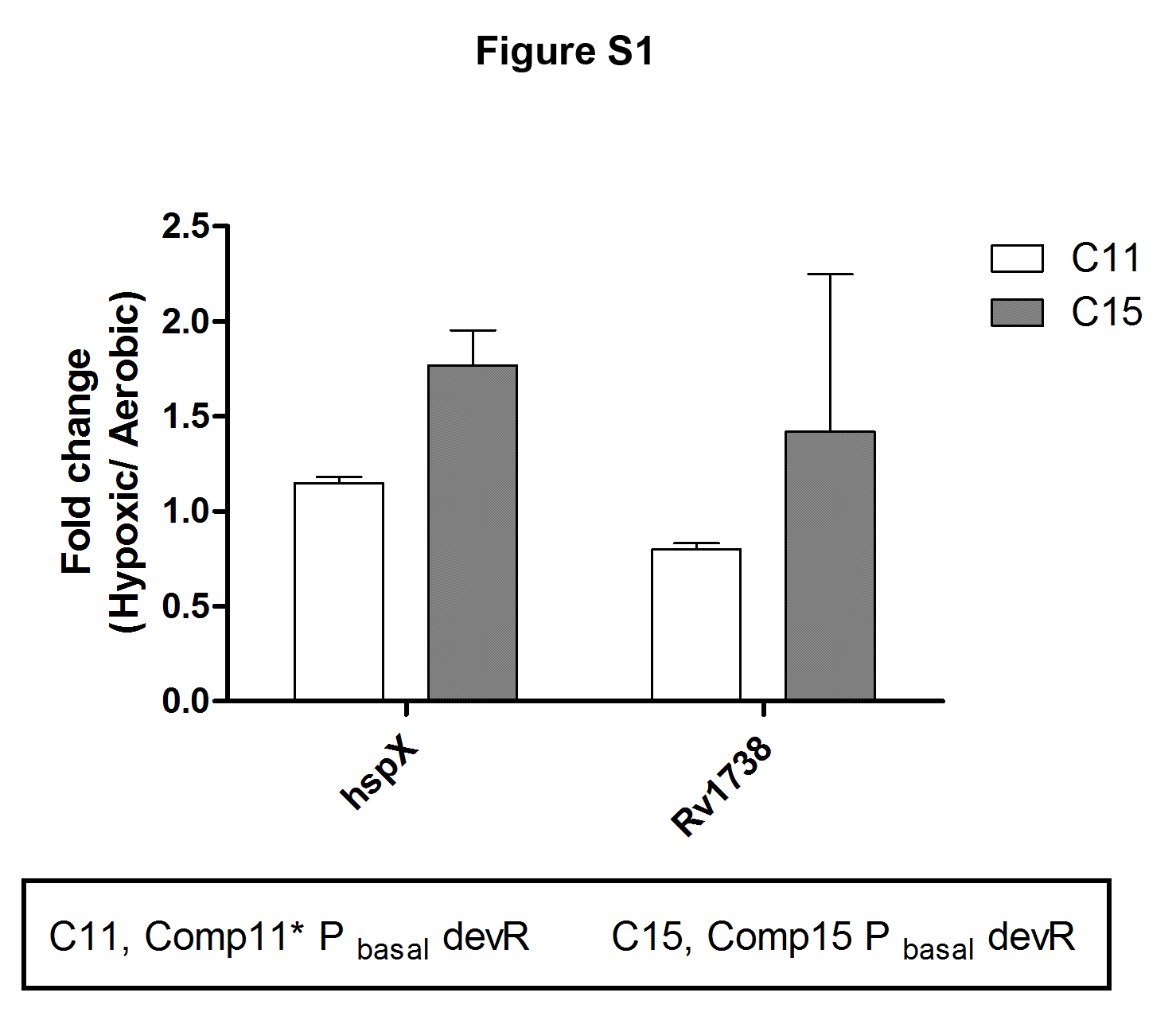

Supplement: Figure S1 — Induction of select DevR regulon genes in Comp11* and Comp15. Fold change in hspX and Rv1738 expression in hypoxic vs. aerobic cultures in Comp11* and Comp15 strains was calculated from normalized transcript levels with respect to 16S rRNA. Mean fold change ± SD determined from three independent cultures is shown. (TIF) [file pone.0035847.s001.tif]

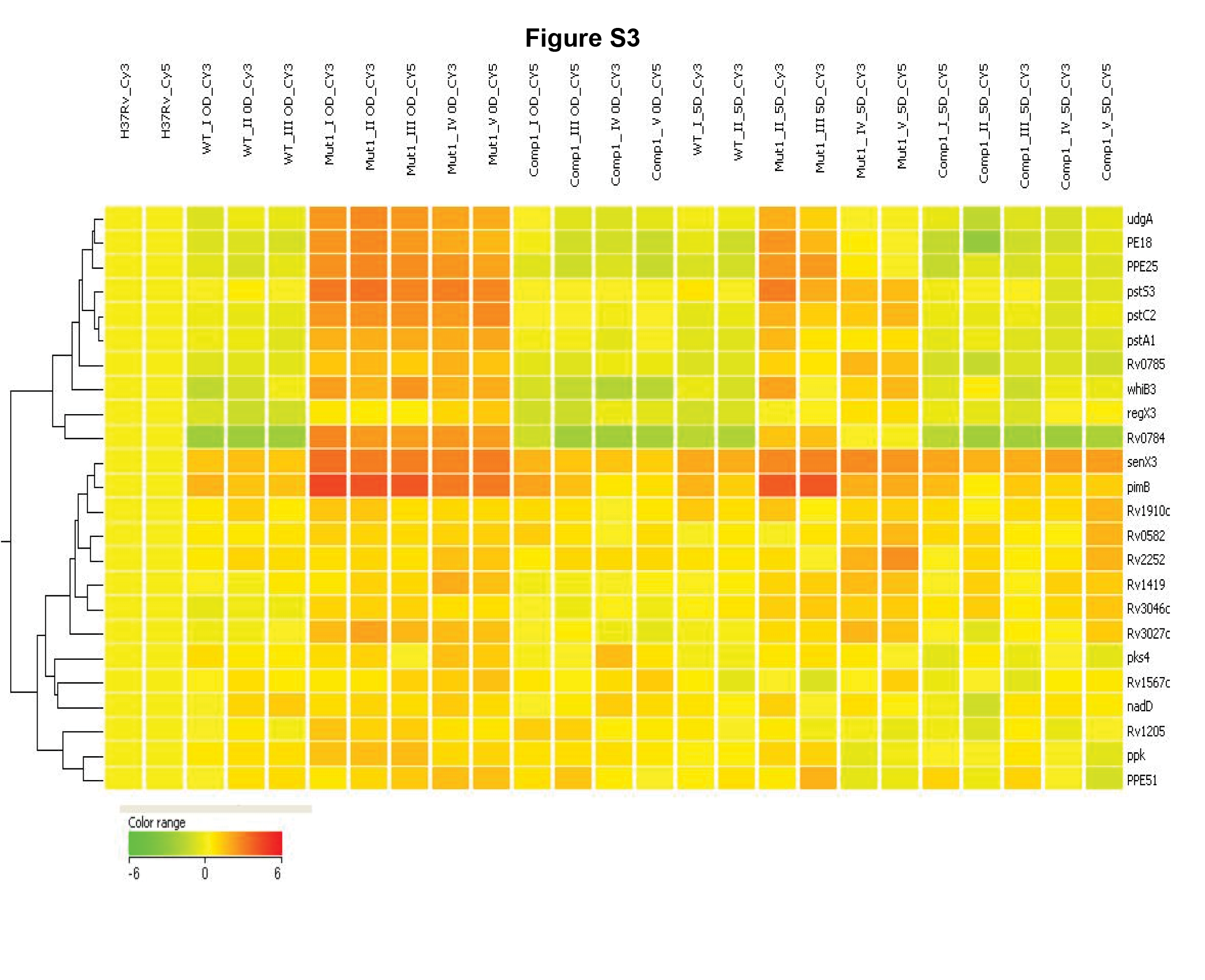

Supplement: Figure S3 — Differential gene expression in M. tb strains. Heat map depicting the fold change in differential gene expression (log base 2) in M. tb strains (2–5 biological replicates). (TIF) [file pone.0035847.s003.tif]
