# Supplementary material for: Appropriate DevR (DosR)-Mediated Signaling Determines Transcriptional Response, Hypoxic Viability and Virulence of Mycobacterium tuberculosis
Source: PLoS One. 2012 Apr 26;7(4):e35847. doi: 10.1371/journal.pone.0035847 (PMC3338549; doi:10.1371/journal.pone.0035847)
Supplement: Table S1 — Virulence of passaged M. tuberculosis strains. (DOCX) [file pone.0035847.s004.docx]

**Table S1. Virulence of passaged *M. tuberculosis* strains.**

| **Weeks** | **Strains** | **Visual scores^#^** | **Spleen weight ratios^** | **Lung weight ratios** |
| --- | --- | --- | --- | --- |
| 6 | WT | 10±5 | 0.43±0.08 | 0.97±0.18 |
|  | Mut1* | 9.8±7.1 | 0.57±0.3 | 0.97±0.58 |
|  | Comp1* | 1.1±1.5* | 0.18±0.02* | 1.19±0.21 |
|  | Comp9* | 11.75±8.5 | 0.484±0.18 | 1.46±0.22 |
|  | Comp11* | 16.8±6.3 | 0.52±0.09 | 1.42±0.27 |
|  | Comp12* | 29.75±7.67* | 1.28±0.32* | 1.77±0.4* |
| 10 | WT | 22.66±13.2 | 1.15±0.31 | 0.89±0.23 |
|  | Mut1* | 10.6±5.27 | 0.61±0.25 | 0.92±0.18 |
|  | Comp1* | 3.8±2.0* | 0.27±0.02* | 0.28±0.08* |
|  | Comp9* | 33.1±11.7 | 0.71±0.22 | 1.2±0.2 |
|  | Comp11* | 22.87±11.8 | 0.637±0.48 | 0.99±0.17 |
|  | Comp12* | 33.83±7.6 | 0.92±0.26 | 1.2±0.16 |
| 13 | WT | 37.2±11.7 | 0.79±0.26 | 1.3±0.13 |
|  | Mut1* | 34±7.6 | 0.65±0.16 | 1.65±0.26 |
|  | Comp1* | 8.5±7.3* | 0.28±0.04* | 0.8±0.04* |
|  | Comp9* | 33.66±7.2 | 0.62±0.13 | 1.52±0.18 |
|  | Comp11* | 35.8±9.28 | 0.67±0.16 | 1.28±0.08 |

# Total lesion scores assigned to spleen, liver, lung and the site of injection along with its draining lymph nodes immediately after death as described and expressed as Mean ± SD [50].

^ Weight ratio = (organ weight/ body weight) × 100.

* P<0.05 in comparison to WT.
