# Supplementary material for: Appropriate DevR (DosR)-Mediated Signaling Determines Transcriptional Response, Hypoxic Viability and Virulence of Mycobacterium tuberculosis
Source: PLoS One. 2012 Apr 26;7(4):e35847. doi: 10.1371/journal.pone.0035847 (PMC3338549; doi:10.1371/journal.pone.0035847)
Supplement: Table S2 — Comparison of our results with previously published microarray analysis. (A) M. tb WT (H37Rv), (B) M. tb dosR ( devR ) mutant. (DOC) [file pone.0035847.s005.doc]

**Table S2. Comparison of our results with previously published microarray analysis**

1. ***M. tb* WT (H37Rv)**

| **Study** | **Hypoxic condition** | **Cut off value** | **Total no. of**  **upregulated genes** | **No. of commonly**  **upregulated genes** | **Total no. of**  **downregulated**  **genes** | **No. of commonly downregulated genes** |
| --- | --- | --- | --- | --- | --- | --- |
| This study | Day 5 standing hypoxia | Up ≥ 1.5-fold  Down ≤ 0.66 | 270 |  | 118 |  |
| Sherman *et al.*, 2001 | 0.2% O_2_ for  2 hrs | Up ≥ 1.5-fold  Down ≤ 0.66 | 360 | 95 | 359 | 42 |
| Park *et al.*,  2003 | 0.2% O_2_ for  2 hrs | Up ≥ 1.8-fold  Down ≤ 0.55 | 240 | 61 | 95 | 8 |
| Voskuil *et al.*, 2004 | Day 4 of NRP,  Wayne model | Up ≥ 1.6-fold  Down ≤ 0.63 | 111 | 69 | 364 | 33 |

1. ***M. tb dosR* (*devR*) mutant**

| **Study** | **Hypoxic condition** | **Cut off value** | **Total no. of**  **upregulated genes** | **No. of commonly**  **upregulated genes** | **Total no. of**  **downregulated**  **genes** | **No. of commonly downregulated genes** |
| --- | --- | --- | --- | --- | --- | --- |
| This study | Day 5 standing hypoxia | Up ≥ 1.5-fold  Down ≤ 0.66 | 297 |  | 435 |  |
| Park *et al.*,  2003 | 0.2% O_2_ for  2 hrs | Up ≥ 1.8-fold  Down ≤ 0.55 | 289 | 56 | 92 | 25 |

**References:**

1. Sherman DR, Voskuil M, Schnappinger D, Liao R, Harrell MI et al. (2001) Regulation of the *Mycobacterium tuberculosis* hypoxic response gene encoding alpha-crystallin. Proc. Natl Acad. Sci. USA 98: 7534–7539.
2. Park HD, Guinn KM, Harrell MI, Liao R, Voskuil MI et al. (2003) Rv3133c/dosR is a transcription factor that mediates the hypoxic response of *Mycobacterium tuberculosis*. Mol Microbiol 48: 833-43.
3. Voskuil MI, Visconti KC, Schoolnik GK (2004) *Mycobacterium tuberculosis* gene expression during adaptation to stationary phase and low-oxygen dormancy. Tuberculosis (Edinb) 84: 218-27.
