# Supplementary material for: Appropriate DevR (DosR)-Mediated Signaling Determines Transcriptional Response, Hypoxic Viability and Virulence of Mycobacterium tuberculosis
Source: PLoS One. 2012 Apr 26;7(4):e35847. doi: 10.1371/journal.pone.0035847 (PMC3338549; doi:10.1371/journal.pone.0035847)
Supplement: Table S3 — Primers used for Real Time Reverse transcriptase-PCR analysis. (DOCX) [file pone.0035847.s006.docx]

**Table S3. Primers used for Real Time Reverse transcriptase-PCR analysis.**

| **Primer** | **Sequence (5' 3')** | **PCR profile** | **Product Size** | **Reference** |
| --- | --- | --- | --- | --- |
|  |  | **Parameters *** |  |  |
| Rv3134c F  Rv3134c R | CTG GCT GGG TCG GCC TTA  GCT GAC CTG GGA GGT TGT CG | 94°C, 30 s  60°C, 30 s  72°C, 30 s | 78 bp | 38 |
| devR f4  devR r3 | CCG ATC TGC GCT GTC TGA TC  GTC CAG CGC CCA CAT CTT T | 94°C, 30 s  52°C, 30 s  72°C, 30 s | 144 bp | This study |
| devS F  devS R | TAC TGA CCG ACC GGG ATC GT  AGA GCC GCT GGA TGA CAT GG | 94°C, 30 s  58°C, 30 s  72°C, 30 s | 60 bp | This study |
| Rv2031c F  Rv2031c R | CGC ACC GAG CAG AAG GA  ACC GTG CGA ACG AAG GA | 94°C, 30 s  56°C, 30 s  72°C, 30 s | 65 bp | 38 |
| Rv1738 F  Rv1738 R | CGA CGA ACA CGA AGG ATT GA  ACA CCC ACC AAT TCC TTT TCC | 94°C, 30 s  56°C, 30 s  72°C, 30 s | 69 bp | -do- |
| fdxA F  fdxA R | TGT CCG GTC GAC TGT ATC TAT GA  GGC AGG CCG GTT TGC | 94°C, 30 s  54°C, 30 s  72°C, 30 s | 91 bp | -do- |
| 16S F  16S R | ATG ACG GCC TTC GGG TTG TAA  CGG CTG CTG GCA CGT AGT TG | 94°C, 30 s  60°C, 30 s  72°C, 30 | 109 bp | -do- |
| senX3 F  senX3 R | CCG AGT TGA TCG AGC TAT CC  GAA ATC GCT TCC GAC ACA AT | 94°C, 30 s  60°C, 30 s  72°C, 30 s | 94 bp | This study |
| regX3 F  regX3 R | TGA CGA CTA CGT GAC CAA GC  CTC ATC TCC GAG TCG TCG TC | 94°C, 30 s  60°C, 30 s  72°C, 30 s | 96 bp | This study |
| pstS3 F  pstS3 R | AAC TAC ACG GCC AAT GGT TC  TCC TTG CTC AGG GGT ACA TC | 94°C, 30 s  60°C, 30 s  72°C, 30 s | 92 bp | This study |
| pstC2 F  pstC2 R | GTT TAA GCA GGG CAA CGT GT  TGT GAC GAT AGG CAG GAT CA | 94°C, 30 s  60°C, 30 s  72°C, 30 s | 91 bp | This study |
| pstA1 F  pstA1 R | CGT GTC AGG CAT CTT GTT GT  GAG GTT GAT GGA GTG GCT GT | 94°C, 30 s  60°C, 30 s  72°C, 30 s | 91 bp | This study |
| whiB3 F  whiB3 R | CGA TCC CAT GCG TTA GAG GT  CAT GGT GCC CTT GAG GAG TA | 94°C, 30 s  60°C, 30 s  72°C, 30 s | 90 bp | This study |
| pimB F  pimB R | TAC GGC ATT CCG ATG ACA G  ATT CCA TTG TCG CTG TGG AC | 94°C, 30 s  60°C, 30 s  72°C, 30 s | 100 bp | This study |
| udgA F  udgA R | GTT TCT GGT GAC CGA TCT GC  ACT TCG GAG ATC GCA TTG AT | 94°C, 30 s  60°C, 30 s  72°C, 30 s | 99 bp | This study |

*40 cycles of amplification
